# Genotype 1 hepatitis E virus infection with acute acalculous cholecystitis as an extrahepatic symptom: a case report

**DOI:** 10.1186/s41182-016-0016-7

**Published:** 2016-06-15

**Authors:** Ken Fujioka, Toshiki Nishimura, Masayuki Seki, Masanori Kinoshita, Nobuyuki Mishima, Shigeo Irimajiri, Masaya Yamato

**Affiliations:** Department of General Internal Medicine and Infectious Diseases, Rinku General Medical Center, Ourai-Kita, Rinku, Izumisano, Osaka, 5988577 Japan

**Keywords:** Case report, Hepatitis E virus, Acalculous cholecystitis, Genotype 1

## Abstract

**Background:**

Hepatitis E virus (HEV) causes an acute viral hepatitis that is transmitted enterically. It is epidemic in Africa, Asia, the Middle East, and Central America. It is known that HEV can cause extrahepatic manifestations. Here, we report the first case of acalculous cholecystitis as an extrahepatic symptom of HEV.

**Case presentation:**

A 24-year-old Japanese woman with no notable past medical history presented with complaints of fever and nausea while she was traveling in Australia; within the previous 2 months, she had also traveled to India and Africa. She visited a local hospital in Australia, and the laboratory tests showed significantly elevated levels of transaminase, so she was checked for viral hepatitis. After excluding hepatitis A, B, and C, as well as other causes of hepatitis, it was revealed that the patient was positive for HEV-IgM. Since she was a visitor to Australia, she was sent back to Japan and was transferred to our hospital. On day 4, the patient complained of right upper quadrant pain. Ultrasonography of the abdomen showed a thickened gallbladder wall without calculi. Acalculous cholecystitis was diagnosed from her course. No antibiotics were administered against it because there was no evidence of bacterial infection. The edematous wall showed significant improvement on day 11 and had returned to normal by day 14. The patient was discharged on day 16 because all of the symptoms had disappeared.

**Conclusions:**

We found that HEV can cause acalculous cholecystitis as an extrahepatic manifestation. In addition, the cholecystitis could be resolved without any antibiotics.

## Background

Hepatitis E virus (HEV) is a non-enveloped, single-stranded RNA virus that can cause acute hepatitis. There are four genotypes of HEV: genotypes 1, 2, 3, and 4 [1]. Genotype 1 and 2 are prevalent in the Indian subcontinent, Asia, the Middle East, and Africa [2]. In addition to the main symptoms of fever, nausea, and jaundice, several extrahepatic symptoms have also been reported, including pancreatitis, arthropathy, aplastic anemia, and Guillain-Barre syndrome [2]. To the best of our knowledge, there have been no reported cases of acalculous cholecystitis as an extrahepatic symptom of HEV.

## Case presentation

A 24-year-old Japanese woman with no notable past medical history presented with complaints of fever and nausea while she was traveling in Australia; within the previous 2 months, she had also traveled to India and Africa. She visited a local hospital in Australia, and the laboratory tests showed significantly elevated levels of transaminase, so she was checked for viral hepatitis. After excluding hepatitis A, B, and C, as well as other causes of hepatitis, it was revealed that the patient was positive for HEV-IgM. Since she was a visitor to Australia, she was sent back to Japan and was transferred to our hospital. During her stay in India and Africa, she ate most of her meals at local restaurants, and she sometimes drank tap water.

Her initial vital signs revealed a blood pressure of 103/64 mmHg, heart rate of 84 beats/min, and body temperature of 36.4 °C. Physical examination revealed jaundice with no tenderness over the right upper quadrant. The chest, extremities, and other systemic examinations were unremarkable. Laboratory investigations revealed an aspartate aminotransferase (AST) level of 1382 U/L, alanine aminotransferase (ALT) level of 2842 U/L, total bilirubin level of 4.8 mg/dL, and direct bilirubin level of 3.9 mg/dL. The test results were all negative for anti-nuclear antibody, anti-mitochondrial antibody, cytomegalovirus IgG and IgM, Epstein-Barr virus, and hepatitis A, B, and C antibodies; but the test for HEV-IgM was positive (Table 1). Her initial ultrasonography of the abdomen revealed splenomegaly (108 × 39 mm) and a small amount of ascites, but no signs of hepatomegaly or an enlarged gallbladder. According to the data above, HEV infection was diagnosed.

**Table 1:** Laboratory data on admission

(Peripheral blood)	(Biochemistry)	(Viral marker)	
WBC 7600/μL	TP 5.4 d/dL	IgM-HA Ab (−)	CMV-IgG (−)
RBC 471 × 104/μL	Alb 3.4 g/dL	HBs Ag (−)	CMV-IgM (−)
Hb 14.3 g/dL	BUN 7.4 mg/dL	HBs Ab (−)	HSV 1 IgG (+)
Hct 41.0 %	Cre 0.55 mg/dL	HBe Ag (−)	HSV 2 IgG (−)
Plt 18.9 × 104/μl	T-Bil 4.8 mg/dL	Hbe Ab (−)	EBV PCR (−)
	D-Bil 3.9 mg/dL	IgM-HBc Ab (−)	
(Coagulation)	AST 1382 IU/L	HBV DNA (−)	(Immunology)
PT 32 %	ALT 2842 IU/L	HCV Ab (−)	ANA (−)
PT-INR 2.07	LDH 555 IU/L	HCV RNA (−)	Anti-mitochondrial Ab (−)
APTT 38.0 秒	*γ*-GTP 103 IU/L		
Fib 186.3 mg/dL	CHE 148 IU/L		
	NH3 31 μg/dL	Dengue IgG (−)	
	CRP 1.2 mg/dL	Dengue IgM (−)	
	Na 138 mEq/L	HEV-IgG (−)	
	Cl 101 mEq/L	HEV-IgM (+)	
	K 3.8 mEq/L		

**Fig. 1:**
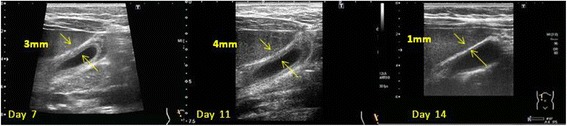
Ultrasonography of the gallbladder. Abdominal ultrasonography revealed a gallbladder without calculi, a thickened gallbladder wall (*arrows*), and perivesical fluid accumulation. These findings resolved by day 14 after admission

She was treated with intravenous fluids with normal saline. On day 4, the patient complained of right upper quadrant pain. Ultrasonography of the abdomen showed 3 mm of a gallbladder wall; moreover, a physical examination detected tenderness over the right upper quadrant and positive Murphy’s sign. Since the levels of transaminase and total bilirubin were gradually declining at that time, the enlarged gallbladder was left untreated, but closely followed up. However, the level of AST was elevated again at 980 U/L on day 7. In addition to the ultrasonographic findings, perivesical fluid accumulation and an edematous gallbladder wall (4 mm) had appeared (Fig. 1). There were no stones in the gallbladder. In addition, there were no other causes of acalculous cholecystitis. Pneumonia, acute pancreatitis, hepatic or subphrenic abscess, and right pyelonephritis were considered for the possible causes but were excluded from the diagnosis due to the fact that no evidence was shown on ultrasonographic findings, urinalysis, and chest X-ray. No antibiotics were administered for the cholecystitis. From day 9, the levels of transaminase and bilirubin began to decline even without the use of antibiotics. Blood culture was negative, and the procalcitonin level was within the normal range. Based on these findings, we assessed the cholecystitis was not caused by bacterial infection and decided not to administer any antibiotics. The edematous wall showed significant improvement on day 11 and had returned to normal by day 14. Since the patient did not complain of abdominal pain and the findings were gradually being recovered, it was not necessary to intervene surgically. The patient was discharged on day 16 because all of the symptoms had disappeared.

**Fig. 2:**
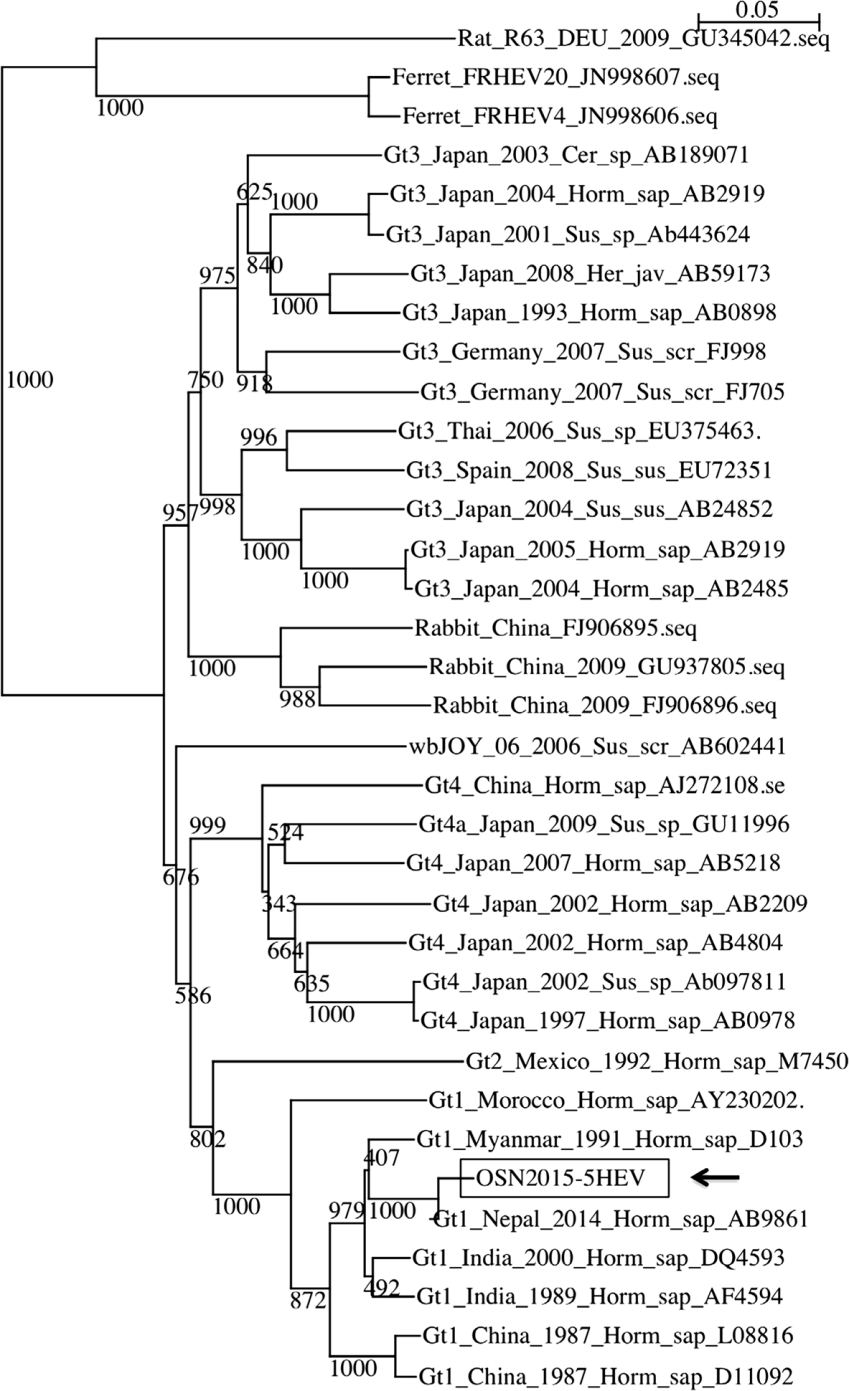
Phylogenetic tree of HEV. OSN2015-5 HEV is the isolate from the patient (*arrow*)

The serum of the patient was tested to identify the genotype of the HEV at the Osaka Prefectural Institute of Public Health, and it was identified to be HEV genotype 1, OSN2015-5 (Fig. 2). It was confirmed by using SuperScript III-one step RT-PCR system with Platinum Taq (Invitrogen).

### Discussion

From this case, two important clinical discoveries were made: (1) HEV can cause acalculous cholecystitis as an extrahepatic manifestation, and (2) it can recover without any antibiotics.

First, HEV can cause acalculous cholecystitis as an extrahepatic symptom. It has been reported that HEV can cause pancreatitis, arthropathy, aplastic anemia, and Guillain-Barre syndrome as extrahepatic symptoms [2, 3]; however, acalculous cholecystitis has not been previously reported as a symptom of HEV. We performed a search of the MEDLINE database for the terms ‘cholecystitis’ and ‘viral hepatitis E’. Three hits were found, but the contexts were unrelated to cholecystitis due to HEV. Hepatitis A virus infection is known to cause acalculous cholecystitis as a rare complication [4]. Although further investigations of a larger number of cases are needed to clarify the matter, it is presumed that hepatitis A virus invades the endothelial cells of the gallbladder and bile duct and induces cell-mediated immunity [4, 5]. However, this is the first case report of acalculous cholecystitis as an extrahepatic manifestation of HEV.

Second, the acalculous cholecystitis due to HEV infection could recover without any antibiotics. On the seventh day after admission, the level of serum AST was increased, and ultrasonography of the abdomen detected a thickened gallbladder wall without calculi and perivesical fluid accumulation; these met the criteria for acalculous cholecystitis [6]. In general, the treatment options for acalculous cholecystitis are antibiotics, drainage, and/or operation. However, none of them were necessary in this case, and the patient recovered completely. According to the clinical course, the cholecystitis was secondary to HEV infection and recovered as the HEV infection resolved.

Humans can be infected by four different genotypes of HEV: genotypes 1, 2, 3, and 4. HEV genotypes 1 and 2 are common and restricted to human [7]. Individuals may become infected with HEV genotypes 1 and 2 from drinking contaminated water, so it was suspected that this patient became infected with HEV from drinking tap water in India. Genotype 1 is prevalent in the Indian subcontinent, Asia, the Middle East, and Africa [2]. The latent period of HEV infection is approximately 6 to 8 weeks [8]. Based on the travel history of the patient, it is possible that she became infected with HEV while she was in India. HEV infection does not affect only developing countries. HEV genotypes 3 and 4 are found in some industrialized countries. In addition, zoonotic transmission to humans is possible with HEV genotypes 3 and 4 [6]. Occasional foodborne outbreaks from the consumption of undercooked meat contaminated with HEV have occurred in Europe, North America, Japan, and New Zealand [9]. Among the different genotypes, genotype 1 HEV infection can cause the most serious disease [10]. As such, it is important to determine the genotype of the HEV infecting a patient to know the prognosis and to identify the source of the infection.

## Conlusion

We found that HEV can cause acalculous cholecystitis as an extrahepatic manifestation. In addition, the cholecystitis could be resolved without any antibiotics. As such, it is necessary to check for the presence of cholecystitis by using abdominal ultrasonography when a patient complains of upper abdominal pain, and laboratory tests show an elevated level of transaminase. In general, the treatment options for acute cholecystitis include antibiotics and operation; however, if the cholecystitis is induced by viral hepatitis, none of these options may be necessary. Further studies are needed to determine how often HEV infection causes cholecystitis.
